# Density‐dependent and density‐independent drivers of population change in Barton Springs salamanders

**DOI:** 10.1002/ece3.4130

**Published:** 2018-05-04

**Authors:** Nathan F. Bendik, Laurie A. Dries

**Affiliations:** ^1^ Watershed Protection Department Austin Texas; ^2^ Present address: Biodiversity Collections University of Texas at Austin Austin Texas

**Keywords:** density‐dependence, density‐independence, endangered species, Eurycea sosorum, karst aquifer, long‐term monitoring, MARSS, time series analysis

## Abstract

Understanding population change is essential for conservation of imperiled species, such as amphibians. Worldwide amphibian declines have provided an impetus for investigating their population dynamics, which can involve both extrinsic (density‐independent) and intrinsic (density‐dependent) drivers acting differentially across multiple life stages or age classes. In this study, we examined the population dynamics of the endangered Barton Springs Salamander (*Eurycea sosorum*) using data from a long‐term monitoring program. We were interested in understanding both the potential environmental drivers (density‐independent factors) and demographic factors (interactions among size classes, negative density dependence) to better inform conservation and management activities. We used data from three different monitoring regimes and multivariate autoregressive state‐space models to quantify environmental effects (seasonality, discharge, algae, and sediment cover), intraspecific interactions among three size classes, and intra‐class density dependence. Results from our primary data set revealed similar patterns among sites and size classes and were corroborated by our out‐of‐sample data. Cross‐correlation analysis showed juvenile abundance was most strongly correlated with a 9‐month lag in aquifer discharge, which we suspect is related to inputs of organic carbon into the aquifer. However, sedimentation limited juvenile abundance at the surface, emphasizing the importance of continued sediment management. Recruitment from juveniles to the sub‐adult size class was evident, but negative density‐dependent feedback ultimately regulated each size class. Negative density dependence may be an encouraging sign for the conservation of *E. sosorum* because populations that can reach carrying capacity are less likely to go extinct compared to unregulated populations far below their carrying capacity. However, periodic population declines coupled with apparent migration into the aquifer complicate assessments of species status. Although both density‐dependent and density‐independent drivers of population change are not always apparent in time series of animal populations, both have important implications for conservation and management of *E. sosorum*.

## 1 INTRODUCTION

Understanding why and how populations change is a fundamental challenge for ecologists (Lande, Engen, & Saether, 2003) and is essential for conservation and management of imperiled and exploited species (Conroy & Carroll, 2009; Williams, Nichols, & Conroy, 2002). Amphibians have experienced declines worldwide (Stuart et al., 2004) highlighting the need to elucidate drivers of population change (Pechmann et al., 1991), and in particular, to quantify the relative contribution of both extrinsic (density‐independent) and intrinsic (density‐dependent) effects (Bancila, Ozgul, Hartel, Sos, & Schmidt, 2016; Greenberg & Green, 2013; Pellet, Schmidt, Fivaz, Perrin, & Grossenbacher, 2006). For example, how populations respond to changes in density can influence their probability of extinction (Morris & Doak, 2002) as well as their response to harvesting or climatic events (Hilborn, Walters, & Ludwig, 1995). However, certain life stages may respond differently to population density (Gamelon et al., 2016) or environmental factors, affecting their contribution to the overall population dynamics. This can be particularly important for groups, such as amphibians, that exhibit complex life cycles (Hellriegel, 2000). Determining how intrinsic and extrinsic factors contribute to amphibian population dynamics may require long time series of abundance data (Berven 2009; Whiteman & Wissinger, 2005; Semlitsch, Scott, Pechmann, & Gibbons, 1996) for multiple size classes or life stages.

**Figure 1:**
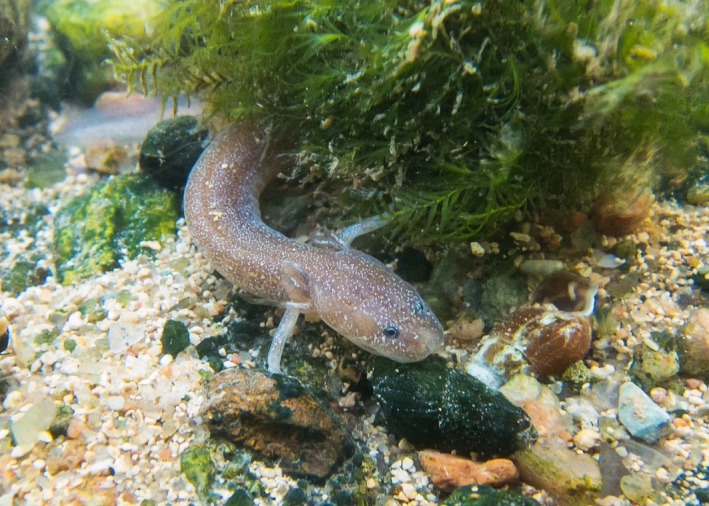
An adult Barton Springs salamander at Eliza Spring (in situ)

Population monitoring of the endangered Barton Springs Salamander (*Eurycea sosorum*; Figure 1) has been ongoing for well over two decades, as the species was initially described from springs that feed a public swimming pool in Austin, Texas (Chippindale, Price, & Hillis, 1993). *Eurycea sosorum* is neotenic and is endemic to four main spring outlets of Barton Springs, as well as the contributing aquifer and springs within its watershed (Devitt & Nissen, 2018). The species is easily observed around spring outlets, but is rarely seen in abundance of more than a dozen individuals at most sites (with the exception of two spring outlets). Threats to *E. sosorum* are exacerbated by its small range and include vulnerability to catastrophic spills over the karst aquifer, declining water quality from development, anthropogenically modified habitat, and declines in water quantity from pumping and climate change (Chippindale & Price, 2005; Chippindale et al., 1993; Stamm et al., 2015; U.S. Fish and Wildlife Service 1997, 2005). While conservation efforts strive to ameliorate habitat loss and disturbance within and around Barton Springs, as well as improve water quality and quantity within the Barton Springs segment of the Edwards Aquifer, a better understanding of how these and other factors influence *E. sosorum* populations is necessary.

Previous analyses of population count data for *E. sosorum* indicate large fluctuations in abundance where a combination of intrinsic and extrinsic factors may be at play, with effects differing among size classes (Gillespie, 2011). Here, we used multivariate autoregressive state‐space (MARSS) models that incorporate both site and size class‐specific effects to fit time series of population counts. MARSS models are advantageous for fitting these data because they can assess both intrinsic (e.g., negative density dependence) and extrinsic interactions among groups (e.g., species or guilds) as well as the contribution of environmental factors to population change (Hampton et al., 2013; Ives, Dennis, Cottingham, & Carpenter, 2003). Additionally, MARSS models can also accommodate missing data (Holmes, Ward, & Wills, 2012) as well as independent estimates of detection error while controlling for temporal autocorrelation inherent in time series data (Ives et al., 2003). Using MARSS models, we quantified the following effects: (1) density‐independent effects related to habitat and hydrologic conditions (seasonality, discharge, algae and sediment cover); (2) size‐specific negative density dependence; and (3) interactions among size classes. We used data from three separate monitoring regimes for our analysis. The primary data set comprises monthly count data collected over a 10‐year period for three size classes of *E. sosorum* from two sites that exhibit the highest known abundances for the species (Eliza and Parthenia springs). To control for observation error within the MARSS model, we use independent estimates of detection probability generated from a capture–recapture study. We then performed a MARSS analysis on a time series of monthly counts collected before our primary data set (using comparable, but not identical, survey techniques) to assess the generality of our findings.

## 2 MATERIALS AND METHODS

### 2.1 Study sites and data collection

**Figure 2:**
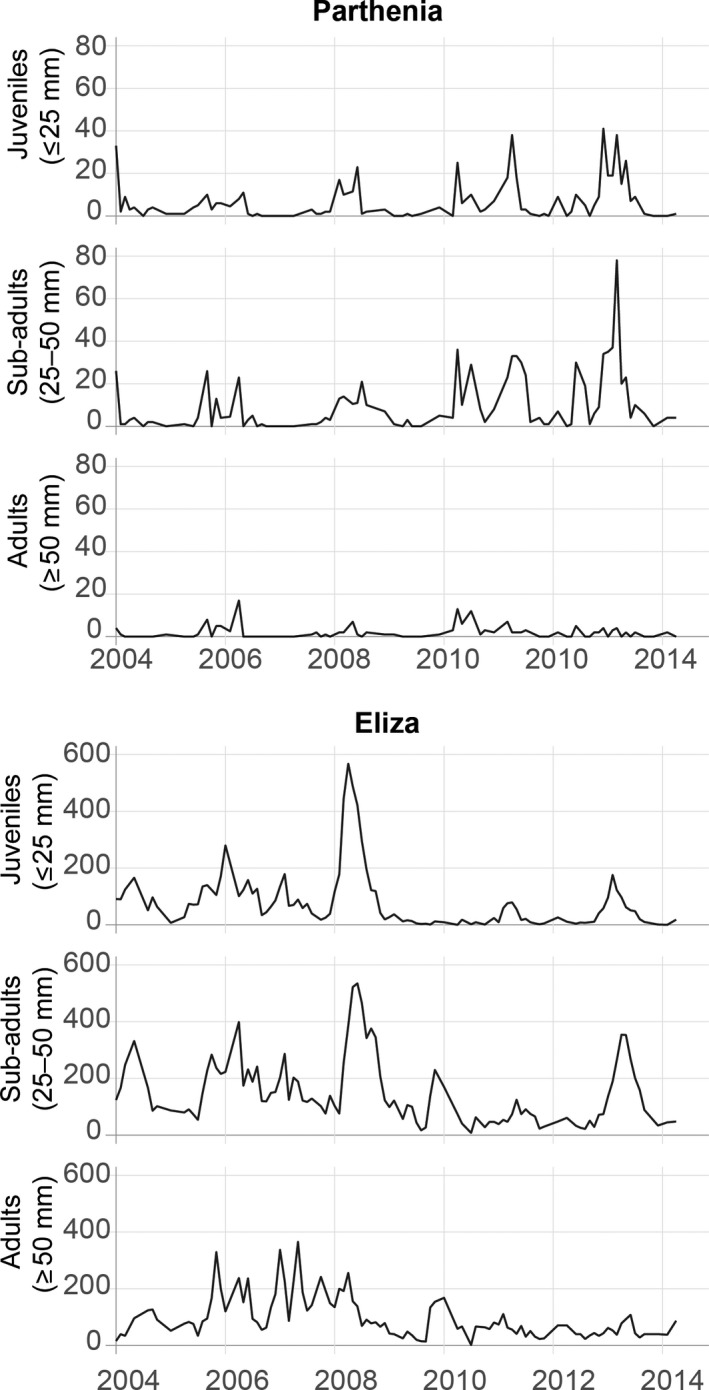
Time series of counts of *E. sosorum* by size class from 2004 to 2014 from Parthenia and Eliza springs. Data from each site are plotted to different *y*‐axes

We performed monthly surveys around Eliza and Parthenia springs, two of the major outlets of the Barton Springs complex in Zilker Park, Austin, Texas, USA. We surveyed at Eliza (74 m^2^) by snorkeling and at Parthenia (158 m^2^) by SCUBA diving. From 2004 to 2014, we enumerated salamanders using a modified drive survey method, whereby all possible cover within the survey area was searched from downstream to upstream and all salamanders were counted. Salamanders were categorized into one of three size classes representing small juveniles (≤ 25 mm; hereafter referred to as “juveniles”), large juveniles and small adults (25–50 mm; “sub‐adults”) and large adults (≥50 mm; “adults”). During each survey, the percent of fine sediment (silt, sand and clay) and percent of filamentous, loosely attached algae covering the substrate was visually estimated; for most surveys, estimates were checked using photographs overlain with a grid. The final time series length was 124 months, with missing data of *n *=* *24 for Eliza and *n *=* *44 for Parthenia, resulting in a total sample size of 180 surveys (Figure 2).

Capture–recapture surveys were conducted at Eliza Spring from 2014 to 2016 on a quarterly basis, following a closed‐population design. This method entails successive sampling over a short enough period where demographic closure (no births, deaths, or migration) of the population can be assumed (Otis, Burnham, White, & Anderson, 1978). For each primary sampling event (*n *=* *7), three surveys were performed, each 1–3 days apart. Surveys were performed as above, with the exception that observers attempted to capture all individuals encountered using handheld nets. All captured salamanders were photographed on a standardized grid background in a water‐filled tray using a Nikon D7100 DSLR and wireless flash. We used the program Wild‐ID (Bolger, Morrison, Vance, Lee, & Farid, 2012) to assist with recognition of individuals based on their unique head markings. The photographic identification method has been validated for a closely related species, *E. tonkawae*, which exhibits a similar characteristic melanophore pattern on its head (Bendik, Morrison, Gluesenkamp, Sanders, & O'Donnell, 2013).

### 2.2 Environmental drivers

Correlations between salamander counts and rainfall at various lags have been reported for subsets of these data (Gillespie, 2011), suggesting the existence of a delay between some environmental driver(s) associated with aquifer recharge/discharge and salamander abundance. To account for this effect, we used mean monthly discharge from Barton Springs (obtained from https://waterdata.usgs.gov/tx/nwis/dv/?site_no=08155500) to compare lags (*k*) of spring discharge up to 15 months (12% of the time series length) with salamander counts using cross‐correlations to help determine which lag period to include as a covariate in the MARSS model. We also included nonlagged spring discharge (*k *=* *0) as a covariate because it is correlated with dissolved oxygen concentration and temperature (Mahler & Bourgeais, 2013), each of which may influence various aspects of spring‐dwelling *Eurycea* ecology, behavior, and physiology (Crow, Forstner, Ostrand, & Tomasso, 2016; Fries, 2002; Woods, Poteet, Hitchings, Brain, & Brooks, 2010).

We included two covariates representing physical effects on the surface substrate where *E. sosorum* lives: percent fine sediment cover (clay, silt and sand) and percent filamentous algae cover. Sedimentation fills interstices in gravel substrate, reducing habitat availability for salamanders (Welsh & Ollivier, 1998). Algae is a food source for macroinvertebrate prey (Allan, 1995), although a high abundance of filamentous algae may be an impediment to salamanders moving along the substrate. Our cover variables included some missing data, as these were not recorded when surveys were also missed.

Because other closely related *Eurycea* inhabiting central Texas springs and streams exhibit seasonal patterns of reproduction and population demographics (Bendik, 2017; Pierce, McEntire, & Wall, 2014), we tested for a seasonal effect on *E. sosorum* abundance to represent un‐sampled covariates. We used a discrete Fourier series to represent seasonality as the following sum: α × cos(2π*d*/12) + β × sin(2π*d*/12), where *d* is an integer representing the month of the year and α and β are model coefficients representing the strength of the seasonality effect.

### 2.3 Multivariate autoregressive state‐space model

We fit *E. sosorum* count data to a first‐order autoregressive MARSS model in log‐space, as shown by the following two equations:

(1) $$x_{t}\;=\;{\mathrm{Bx}}_{t-1}+u+{\mathrm{Cc}}_{t-k}+w_{t},\;\text{where}\;w_{t}∼\text{MVN}\left(0,\;S\right)$$

(2) $$y_{t}\;=\;x_{t}+v_{t}\;\text{where}\;v_{t}∼\text{MVN}\left(0,\;R\right).$$

The process (state) part of the model is given in Equation (1), where **x**_*t*_ is the *m *×* *1 vector of salamander log‐abundances for each of *m* size classes for each site at time *t*,** B** is a *m *×* m* interaction matrix whose elements *b*_*ij*_ describe the effect of class *j* on the per capita growth rate of class *i*,** u** is the *m *×* *1 vector of the long‐term population growth rates (*u*_*i*_) for each class, **c**_*t−k*_ is the *p *×* *1 vector of covariates at time *t* minus lag *k*, and **C** is the *m *×* p* matrix whose elements *c*_*ij*_ describe the effect of covariate *j* on class *i*. The vector of process errors (**w**_*t*_) is assumed to be serially uncorrelated and drawn from a multivariate normal distribution (MVN) with a mean of 0 and covariance matrix **S**. Equation (2) describes the observation component of the MARSS model, where **y**_*t*_ is the *n *×* *1 vector of log counts (*y *+* *1) for state variable **x**_*t*_. The vectors of process error (**w**_*t*_) and observation error (**v**_*t*_) are assumed serially uncorrelated and drawn from multivariate normal distributions (MVN) with means of zero and covariance matrices **S** and **R**, respectively.

We incorporated density‐independent factors (i.e., environmental effects) into the MARSS model through covariates **c**_*t−k*_. Because **c** cannot contain missing data, we predicted missing values using a MARSS model of the covariate time series and replaced the missing values with the predicted values (Figure S1).

In addition to covariates, environmental stochasticity is incorporated into the process error term, **S**. We considered the following five model structures for process error: (1) equal variance and covariance (*K* = 2 variance‐covariance parameters); (2) site‐specific variance and covariance (*K* = 4); (3) site‐specific variance and covariance with between‐site covariance (*K* = 5); (4) size‐specific variance and covariance (*K* = 6); (5) size‐ and site‐specific variance and covariance (*K* = 12).

Negative density dependence and interactions between size classes are represented by parameters of the **B** matrix from Equation (1). We considered negative intra‐class density dependence (hereafter, simply “density dependence”) and between size class interactions, represented by the following matrix:

$$\left[\begin{array}{cccccc} b_{j\to j}^{P} & b_{s\to j}^{P} & b_{a\to j}^{P} & 0 & 0 & 0\\ b_{j\to s}^{P} & b_{s\to s}^{P} & b_{a\to s}^{P} & 0 & 0 & 0\\ 0 & b_{s\to a}^{P} & b_{a\to a}^{P} & 0 & 0 & 0\\ 0 & 0 & 0 & b_{j\to j}^{E} & b_{s\to j}^{E} & b_{a\to j}^{E}\\ 0 & 0 & 0 & b_{j\to s}^{E} & b_{s\to s}^{E} & b_{a\to s}^{E}\\ 0 & 0 & 0 & 0 & b_{s\to a}^{E} & b_{a\to a}^{E} \end{array}\right]$$

Subscripts on the *b*s represent small juveniles (*j*), sub‐adults (*s*), and adults (*a*), with the first representing its effect on the second in the following month. Superscripts indicate site (P = Parthenia, E = Eliza). The diagonal elements represent the effects of density dependence (*b *=* *1 implies density independence, 0 < *b *<* *1 implies undercompensation, *b *<* *0 implies overcompensation; Hampton et al., 2013). The off‐diagonal elements (among *b*s for each site) indicate the strength of inter‐class competition, cannibalism, and recruitment into the next size class. In general, we expected larger size classes to have negative effects on smaller size classes due to cannibalism. Conversely, we expected a positive influence of smaller size classes on larger ones because of growth and recruitment into the next class. We assumed that the effects of juveniles on adults were negligible and fixed that interaction to zero. Juveniles would take much more than 1 month to recruit to adults (thus negating a recruitment effect at the resolution of our data) and are unlikely to compete with adults for the same prey based on differences in gape size. Because we could only observe individuals at the surface (and not the subsurface) during surveys, **B** includes the per capita rate of population growth and factors in movement of individuals between the surface and sub‐surface. Thus, density‐dependent effects in **B** include population growth as well as the spatial distribution of individuals. We did not consider interactions between sites, as we believe migration is infrequent, and preliminary capture–recapture results thus far support this view (City of Austin, unpublished data). Therefore, we fixed elements representing between‐site interactions within **B** to zero.

We fixed the diagonal elements of covariance matrix **R** based upon independent estimates of observation error variance from capture–recapture data collected from 2014 to 2016. While the elements of **R** can be estimated directly from the count data, this can result in higher variance in the **B** estimates (Hampton et al., 2013) or problems with parameter identifiability (Knape, 2008); even rough estimates of **R** are preferred over direct estimation (Ives et al., 2003). We used program MARK (White, 2015) and package RMark (Laake, 2013) in program R (R Development Core Team, 2016) to fit capture–recapture data to closed‐population models (Otis et al., 1978) allowing for time variation in capture probability by size class. The lognormal variance of observation error from all estimates of capture probability was 0.02, which we used to fix the diagonal elements of **R** (because the MARSS model is expressed in log‐space). In doing so, we assumed that variance in observation error measured from the capture–recapture data at Eliza from 2014 to 2016 was equivalent for count surveys at both sites over the course of our study. We also assumed that other nonprocess errors included in **R** were small.

### 2.4 Model selection and validation

We fit models using the MARSS package (Holmes et al., 2012) in program R. Prior to including covariates, we used condition number tests (Montgomery, Peck, & Vining, 2006) and confirmed the absence of substantial multicollinearity (Table S1). To aid with numerical estimation and interpretation of coefficients among covariates that differ in magnitude, we standardized all variables to dimensionless units (*z*‐scores) prior to analysis by subtracting the mean and dividing by the standard deviation, so that coefficients are directly comparable (Hampton et al., 2008).

We compared models with Akaike's information criterion modified for state‐space models using a parametric bootstrap procedure (AIC*b*; Cavanaugh & Shumway, 1997; Ward et al., 2010), as implemented in R package MARSS. Model selection was performed using three phases to avoid fitting a large number of models (representing many combinations of hypotheses), which would increase the possibility of obtaining a spurious result. In the first phase, we fit the most general model with and without a long‐term growth rate, *u*. In the second phase, we proceeded with the AIC*b* best model for *u* and compared sub‐models with differing error structures as described above. In the third phase, we fit a suite of 32 models, which included all possible combinations of covariates (including a no‐covariate model), excluding interactions. We allowed the effect of covariates to vary between sites and between juveniles and sub‐adults/adults. We combined covariate effects for sub‐adults and adults to control parameter bloat and avoid overfitting; our goal was to keep the number of parameters to ~10 data points per parameter following the common rule of thumb. This reflects our assumption that if covariate effects vary among size classes, they will be most dramatic between juveniles and larger classes. As a diagnostic check of our phase‐based approach to model selection, we also ran our best model with intra‐class effects only (with off‐diagonal elements of **B** set to zero) and with a nonzero long‐term growth rate (*u*) with **B** set as an identity matrix (density independence). We calculated AIC*b* values for each model from 1,000 iterations and confidence intervals from 1,000 parametric bootstraps. We used model averaging to graphically represent species interactions and covariate effects.

We determined the adequacy of model fit by examining residuals from the top AIC*b* model and did not find any extreme deviations from normality or other patterns indicative of poor fit (Figures S2–S7). Although there was a strong lag‐1 autocorrelation of the observation residuals for Eliza, increasing the observation variance to 0.06 for juveniles and sub‐adults and 0.12 for adults mostly ameliorated the problem. This had no substantial effects on the coefficient estimates or their standard errors. Therefore, we present results with the calculated observation variance of 0.02.

To cross‐validate our findings, we also analyzed out‐of‐sample count data collected at Parthenia during the prior decade, from 1993 to 2003. Surveys generally occurred monthly, although some months included multiple surveys while other months were missed. We averaged values for surveys within the same month, resulting in a final sample size of 90 surveys. Data were collected from the same area as our primary dataset, but a combination of transects, quadrats, and judgment sampling was used instead of an exhaustive search, and salamanders were categorized into two size classes instead of three, <25 mm TL and >25 mm TL. We examined cross‐correlations with discharge (as above) and applied our most general model, including seasonality, discharge, and lagged discharge as covariates on both size classes (fine sediment and filamentous algae cover data were unavailable for this period). Although some survey data for Eliza were also available, these were too sparse for a comparable analysis.

## 3 RESULTS

In general, more complex models with intra‐ and inter‐size class interactions, several covariates, and a complex error structure were favored over simpler models in our analysis. During the first phase of model selection, the most general model with a long‐term growth rate performed poorly compared to one without it (ΔAIC*b* = 24). Using this structure for the next phase, the size‐ and site‐specific variance–covariance structure was the best‐supported model of process error (ΔAIC*b* = 19 for the next best model). For the final phase of model selection, we compared models with all possible combinations of covariate effects (excluding interactions), which resulted in two models that accounted for >99% of the total AIC*b* weight within the final model set (Table 1). Modifying the best model to exclude inter‐class interactions and density dependence altogether resulted in substantially worse models (ΔAIC*b* = 32 and 212, respectively). Parameter estimates were similar among the top five models (Table S2).

**Table 1:** The top five of 32 multivariate autoregressive state‐space models comparing the importance of algae cover, sediment cover, spring discharge, season, and a 9‐month lag of spring discharge as predictors of relative abundance of small juvenile (≤ 25 mm TL), sub‐adult (25–50 mm TL), and adult (≥50 mm TL) *E. sosorum* at Parthenia and Eliza springs

Model covariates	AIC*b* a	ΔAIC*b* b	ω^c^	−2log (*L*)	*K* d	Evidence ratio
Sediment, lagged discharge	1,034.35		0.86	928.50	40	
Sediment, algae, lagged discharge	1,038.12	3.77	0.13	917.34	44	6.6
Sediment, seasons, lagged discharge	1,045.05	10.70	0.00	912.27	48	32.0
Sediment, algae, season, lagged discharge	1,048.76	14.40	0.00	899.99	52	1,342.1
Lagged discharge	1,049.04	14.69	0.00	955.48	36	1,546.1

Lagged spring flow was only included as a predictor of small juvenile abundance.

a Bootstrap Akaike's Information criterion.

b Difference between the AIC*b* value for candidate model and the AIC*b* value of the best‐approximating model.

c AIC*b* weights. Probability that candidate model is the best‐approximating model within the model set.

d Number of parameters estimated in each model.

**Figure 3:**
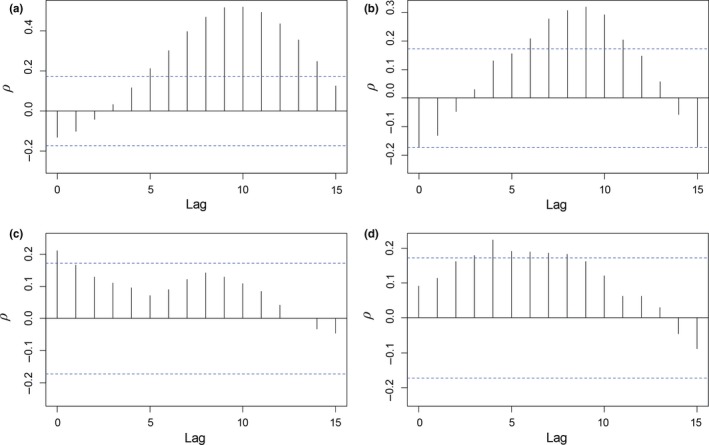
Cross‐correlation between counts of *E. sosorum* and mean monthly discharge of Barton Springs at lags *k *=* *0–15 months. (a) Juvenile (≤ 25 mm TL) abundance at Eliza; (b) Juvenile abundance at Parthenia; (c) Adult (≥ 50 mm TL) abundance at Eliza; (d) Adult abundance at Parthenia. Dashed lines represent 95% confidence limits (calculated at lag *k *=* *1)

Discharge was correlated with juvenile abundance for a series of lags from 5 to 14 months for Eliza and 6 to 12 months for Parthenia, with peaks around 9 months at both sites (Figure 3a,b). Conversely, correlations between adult abundance and discharge were weaker and inconsistent between sites (Figure 3c,d). We therefore used the discharge lag of 9 months as a predictor of juvenile abundance (hereafter: “lagged discharge”). We did not find evidence of significant lagged effects for the other covariates examined.

**Figure 4:**
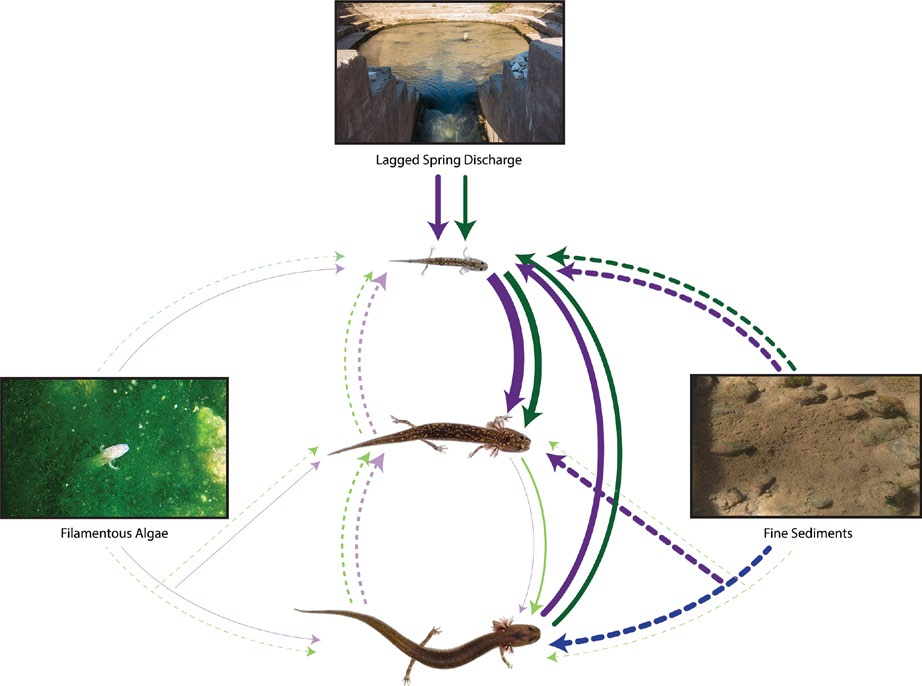
Model‐averaged size class interactions and environmental drivers of *E. sosorum* abundance at Eliza (green lines) and Parthenia (purple lines) from the top two AIC *b* models (total weight = 0.99). Line weights represent the size of model‐averaged coefficients; dashed and solid lines indicate negative and positive relationships, respectively. Lighter colored lines indicate coefficients that included zero within their 95% confidence intervals

Environmental variables had similar effects among sites, but varied by salamander size class. The effect of lagged discharge and sediment cover both appeared in all the top models (Table 1). Lagged discharge had a positive effect on juvenile abundance at both sites (Figure 4). Sediment had a negative effect at both sites and for all size classes, but more strongly affected juveniles (Figure 4). Seasonality and nonlagged discharge were not important predictors of relative salamander abundance based on the AIC*b* rankings (Table 1). Algae cover was marginally important according to the AIC*b* rankings and exhibited varying effects among size classes and sites; coefficients of algae cover were negative for Eliza and positive for Parthenia (Figure 4), although each of the four coefficients included zero within their 95% confidence intervals. Additionally, because the model with algae cover was not weighted heavily (Table 1), the model averaged values were small compared to the other coefficients (Figure 4).

Density dependence was stronger at Parthenia compared to Eliza across all size classes (0.25, 0.12, and 0.20 for juveniles, sub‐adults, and adults, respectively, at Parthenia compared to 0.63, 0.65, and 0.61 at Eliza). In general, inter‐class interactions were similar between sites. Adults at time *t* − 1 had a negligible effect on sub‐adults at time *t*, but in contrast to our prediction, showed a positive relationship with juveniles (Figure 4). Sub‐adults exert a negative effect on juveniles, although 95% confidence limits for these estimates included zero (Figure 4). The effect of sub‐adults on adults was positive, but weak. Evidence of recruitment was apparent from the positive juvenile to sub‐adult interaction (Figure 4), indicating growth of individuals ≤25 mm TL into the next larger size class. All parameter estimates and their 95% confidence intervals for coefficients of the **B** and **C** matrix are provided in the Table S2.

Process error variance estimates represent the unexplained environmental variability present in each time series. Error variances were highest for the adult size class for both sites, indicating that the MARSS model performed poorly in attributing variation in adult abundance to specific drivers in **C** or **B** (e.g., 0.87, 0.57 and 0.48 for Parthenia adults, sub‐adults, and juveniles, respectively). Error covariances quantify the shared response of each size class to environmental stochasticity from an unmodeled driver. Changes in adult and sub‐adult abundance were more strongly correlated (0.49 and 0.25 at Parthenia and Eliza, respectively) compared to changes in juvenile abundance with either size class (e.g., 0.34 and 0.11 for the juvenile to sub‐adult correlation at Parthenia and Eliza, respectively), suggesting that adults and sub‐adults respond more similarly to environmental changes.

The out‐of‐sample data analyses corroborated our findings from the primary (“within‐sample”) analyses. The cross‐correlation revealed a peak between juvenile abundance and lagged discharge at 9 months (Figure S8), which is the same as the within‐sample results (Figure 3). Lagged discharge was the most important environmental predictor of salamander abundance in the out‐of‐sample MARSS analysis (although we were not able to test the effect of sediment or algae); coefficient values for the other covariates were smaller and their confidence intervals each included zero (Table S2). Additionally, we found evidence of recruitment from juveniles to sub‐adult/adults as well as a positive effect of sub‐adults/adults on juveniles, also consistent with our main findings (Figure 4).

## 4 DISCUSSION

The effects of density‐independent factors in regulated populations are not always apparent in time series analyses of animal abundance data (Bancila et al., 2016; Knape & de Valpine, 2011; Rotella et al., 2009). This may be because of a lack of detailed a priori knowledge of a system, for example, when fitting variables that only indirectly influence a population (Knape & de Valpine, 2011). Although we had limited information about the mechanisms influencing population dynamics of *E. sosorum* or similar species (e.g., only observational data are available), we detected strong effects of some exogenous and endogenous drivers, and these were corroborated by a large out‐of‐sample data set. Our results demonstrate that both density dependence and density independence can have important implications for conservation of endangered species, such as *E. sosorum*, by identifying potential mechanisms that limit and regulate their populations.

Direct density‐dependent feedback was present for all size classes of *E. sosorum*, although differences in the strength of density dependence between sites overshadowed differences among size classes. Our results are consistent with a growing body of literature demonstrating the importance of density‐dependent regulation in amphibians (Greenberg & Green, 2013; Salvidio, 2009) which is not necessarily restricted to the larval or juvenile stages (Altwegg, 2003; Berven, 2009; Harper & Semlitsch, 2007; Patrick, Harper, Hunter, & Calhoun, 2008). Although it is difficult to infer the mechanism of density dependence without experiments, different ecological contexts at each site may explain the differences in the strength of the effect we observed. For example, predation‐induced density dependence (Anderson, 2001; Hixon & Jones, 2005) might be a regulating factor at Parthenia, where predatory centrarchid fishes are prevalent, but not at Eliza, where they are generally absent. The degree of density dependence estimated is also similar among size classes, suggesting that if it is predation induced, this could indicate the predator has similar selectivity among salamander size classes. Sediment can be a regulating factor as well, because it can negatively affect the amount of available habitat and prey for salamanders. Additionally, the underlying mechanisms of density dependence may vary among size classes. Because adults are the strongest swimmers, they may move against the flow into areas where densities are lower to avoid competition or predation. Juvenile *Eurycea* have more difficulty moving against strong currents (Barrett, Helms, Guyer, & Schoonover, 2010) such as those issuing from spring openings during wetter conditions. Unable to disperse as easily as adults, the juvenile *E. sosorum* could be more susceptible to density‐dependent survival, similar to larvae of pond‐breeding amphibians (Wilbur, 1997).

We did not find strong evidence for competition and cannibalism, which can cause complex dynamics in size‐structured populations (Claessen, de Roos, & Persson, 2004). Cannibalism could explain the negative adult to sub‐adult and sub‐adult to juvenile interactions, although these effects were relatively weak. In contrast to our expectations, adults had a strong, positive influence on juveniles. Reproduction is not a likely cause, as it occurs on a longer time scale than the time series interval of 1 month (Cantu, Crow, & Ostrand, 2016). In MARSS models, correlation with an unmodeled factor can result in interactions within the **B** matrix (Hampton et al., 2013), which may be the case here. The unexplained correlation between adults and juveniles could be the result of some external driver common to both size classes that results in a lagged correlation in their abundance. For example, this may occur due to temporal variation in prey availability (Gillespie, 2013), a factor we did not measure.

Recruitment was evident from the strong, positive juvenile to sub‐adult relationship, although we did not observe this effect from sub‐adult to adult. Smaller individuals grow faster; therefore, recruitment should be more apparent from month‐to‐month changes in abundance between the smaller size classes, whereas recruitment to the adult size class takes longer. Because we used monthly time intervals with a single lag period, it is possible we are missing time‐delayed effects in the population dynamics, such as delayed density dependence or other interactions. However, there is a trade‐off between using short or long sampling intervals, the latter of which is more likely to confound indirect interactions (e.g., those with an unmodeled species; Ives, Carpenter, & Dennis, 1999).

Monthly changes in surface substrate conditions appear to be an important factor limiting abundance of *E. sosorum*. Salamander abundance was negatively associated with sediment cover, as suggested by Dries and Colucci (2018), and this effect was most pronounced for juveniles. In contrast to sediment, the effect of filamentous algae differed by site and it did not appear in the top model. Excess sediment is a pollutant in the Barton Springs ecosystem (U.S. Fish and Wildlife Service, 2005) and is driven by the local hydraulic controls at each spring site, as well as the sediment supply to Barton Springs (from allochthonous inputs to the aquifer; Mahler & Lynch, 1999). Man‐made impoundments at each site strongly influence local hydrology (Dries & Colucci, 2018) while the sediment supply is driven by storm events over the aquifer and possibly seasonal conditions (Mahler & Lynch, 1999). We cannot rule out that we observed fewer salamanders because of high sediment or algae cover, as these can impede detection in the field. However, interstitial spaces that are an important habitat feature for stream‐dwelling salamanders can be filled with sediment (Martin, Harris, Collums, & Bonett, 2012; Welsh & Ollivier, 1998). Sediment may also negatively influence their invertebrate prey and have other, far‐reaching impacts on stream ecology (Wood & Armitage, 1997).

We did not find evidence for an immediate effect of spring discharge, suggesting that other correlated factors (e.g., flow velocity, dissolved oxygen concentration) do not directly affect salamander abundance at these sites (although flow velocity may be an important factor at other sites; Dries & Colucci, 2018). Similarly, there was no effect of seasonality, in contrast to other closely related central Texas *Eurycea* with seasonally dependent dynamics (Bendik, 2017; Pierce et al., 2014). Seasonal changes in light availability and air temperature are probably less likely to influence large systems with potentially vast subterranean habitat such as the Barton Springs segment of the Edwards Aquifer, in contrast to shallower groundwater systems.

The subterranean niche likely plays a crucial role in population dynamics of *E. sosorum*. Surface populations are unlikely to persist without underground dispersal, given the occurrence of zero or near‐zero counts followed by pulses of increased abundance (this pattern is particularly evident at Parthenia Spring; Figure 2). Additionally, the correlation between juvenile abundance and lagged discharge suggests that processes occurring within the aquifer and aquifer recharge zone are critical for reproduction. The lagged correlation between aquifer discharge and salamander abundance is consistent with prior analyses of these data suggesting lags related to rainfall at 4 to 11 months (Gillespie, 2011). Similarly, pulses in juvenile abundance in nearby *E. pterophila* have been documented approximately four months following storm events (figure 1 in Krejca, McHenry, McDermid, Adcock, & Forstner, 2017). Here, the lagged correlation was strongest at nine months, which was consistent across both data sets encompassing a 21‐year period. Nine months is longer than required for just reproduction and growth based on estimates in captivity (Cantu et al., 2016). The additional delay between discharge and changes in juvenile abundance may be related to ecosystem processes. Storm events introduce organic matter into the subterranean ecosystem (Mahler & Lynch, 1999), which is an important limiting resource in some karst streams (Simon & Benfield, 2002). In these systems, dissolved organic matter incorporated by microbes (e.g., in epilithic biofilms) ultimately transfers through the food chain to higher order predators (Simon, Benfield, & Macko, 2003). For example, neotenic *Gyrinophilus* salamanders exhibit higher density and biomass in cave streams with high organic matter (Huntsman, Venarsky, Benstead, & Huryn, 2011). Therefore, organic matter flushed into the Barton Springs aquifer from storm events may result in higher reproductive output for *E. sosorum* via energy transfer through the food web.

### 4.1 Conservation and management

Populations that reach carrying capacity are less likely to go extinct compared to unregulated populations far below their carrying capacity (Morris & Doak, 2002). Therefore, a pattern of negative density dependence may be a positive sign for *E. sosorum* because it indicates that populations can reach their carrying capacity. However, the species may still be at risk of extinction if habitat is too small or low quality to support evolutionarily viable populations. Additionally, periodic declines resulting in zero or near‐zero abundances complicate assessments of species viability. These periods of very low abundance may indicate extirpation, followed by recolonization, in which the surface habitat acts as a population sink (Pulliam, 1988). This effect may be exacerbated by the fragmented state of surface habitat, which is separated by man‐made impoundments that inhibit movement among springs. Alternatively, individuals may shift between observable and unobservable states (i.e., between the surface and subsurface) and persist as part of the local population; for example, as adults move underground to lay eggs (as we rarely encountered eggs at the surface). Thus, an important question in further understanding both the population dynamics and the conservation status of *E. sosorum* is whether (or what proportion of) the population comprises individuals returning to the surface versus new immigrants recolonizing a population sink. Efforts are underway to quantify temporary emigration using capture–recapture methods (Kendall, Nichols, & Hines, 1997).

Our results have implications for both in situ and ex situ conservation. Biologists maintain surface habitat by periodically flushing fine sediments by hand during and between surveys (Dries et al., 2013); this is intended to mitigate eventual build‐up that results in loss of interstitial habitat for salamanders and their prey. Although the estimate of percent sediment cover we used here is a coarse metric, the strength of this relationship and its consistency between sites suggests efforts to mitigate and reduce fine sediment build‐up should continue. Restoring degraded or lost habitat is also a key to conservation of *E. sosorum*. Density‐dependent population growth indicates these populations are limited by carrying capacity. Therefore, populations should respond favorably to habitat expansion if it results in an increase in carrying capacity. Density‐dependent feedback also indicates some portion of mortality is compensatory, and therefore, may be offset by periodic collections to augment ex situ captive‐breeding programs. Although calculation of compensatory reserve (the capacity of a population to offset variation in mortality; Rose, Cowan, Winemiller, Myers, & Hilborn, 2001) is beyond the scope of this study, it appears that a small number of juveniles may be periodically collected during population booms without substantial harm to wild populations. Regional efforts to protect the Barton Springs aquifer are crucial as well (U.S. Fish and Wildlife Service, 1997, 2005). While the need to protect water quality and quantity for the conservation of *E. sosorum* has long been recognized (Chippindale et al., 1993), we suggest recharge of both water and organic matter to the aquifer may trigger reproduction in *E. sosorum*. This highlights the importance of ongoing efforts to protect natural aquifer recharge (e.g., through land conservation) as well as the need to further investigate the underlying ecological mechanisms linking recharge to reproduction.

## CONFLICT OF INTEREST

None declared.

## AUTHOR CONTRIBUTIONS

LD designed the primary field study; NB designed the capture‐recapture study; NB and LD collected data; NB analyzed the data; NB led the writing of the manuscript. Both authors contributed critically to the drafts and gave final approval for publication.

## DATA ACCESSIBILITY

Data are available from the City of Austin's Open Data Portal.

Count and habitat data: https://data.austintexas.gov/Environment/Barton-Spring-Salamander-Counts-and-Covariates/brj7-e355

Capture–recapture histories: https://data.austintexas.gov/Environment/Barton-Springs-Salamander-Eliza-Spring-Capture-Rec/jcks-vz7q/data?firstRun=true
