# Supplementary material for: Density‐dependent and density‐independent drivers of population change in Barton Springs salamanders
Source: Ecol Evol. 2018 May 4;8(11):5912–23. doi: 10.1002/ece3.4130 (PMC6010705; doi:10.1002/ece3.4130)
Supplement: Supplementary file 1 [file ECE3-8-5912-s001.docx]

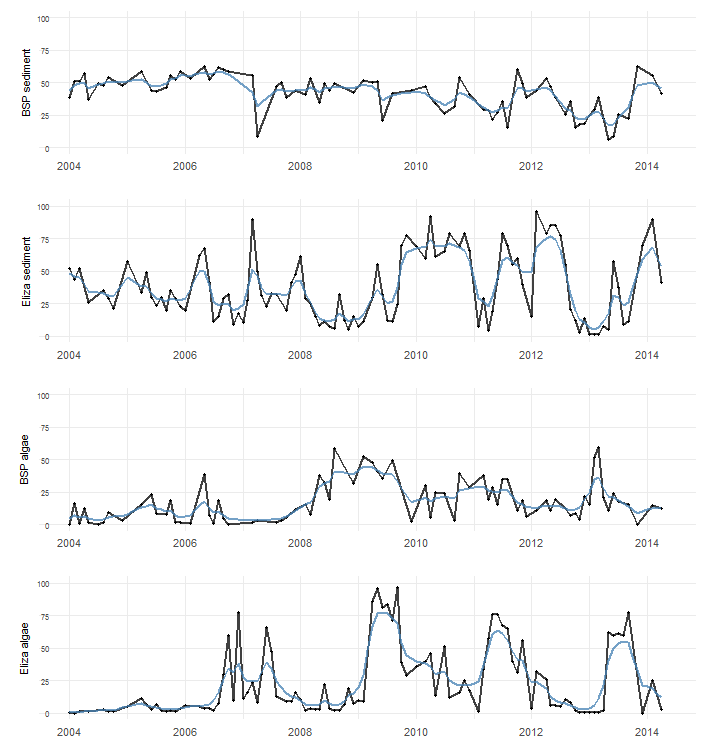


Figure S1. Covariate data (black lines) verses predicted values from MARSS model (blue lines). The predicted values were used to replace only missing values in the covariate data sets for the analysis of salamander counts.

Table S1. Condition index analysis to assess multicollinearity among potential continuous predictor variables.

|  |  |  | **Eigenvectors** | | | | | | | |
| --- | --- | --- | --- | --- | --- | --- | --- | --- | --- | --- |
| **Number** | **Eigenvalue** | **Condition Index** | Discharge | Parthenia Sediment | Parthenia Algae | Eliza Sediment | Eliza Algae | cos(2π*d*/12) | sin(2π*d*/12) | Lagged Discharge |
| 1 | 213.12 | 1.00 | 0.63 | 0.07 | 0.39 | 0.16 | -0.36 | 0.09 | -0.06 | 0.53 |
| 2 | 177.29 | 1.10 | 0.19 | 0.26 | -0.56 | -0.50 | -0.12 | 0.42 | 0.32 | 0.19 |
| 3 | 94.28 | 1.50 | -0.45 | -0.19 | 0.22 | -0.54 | -0.53 | -0.01 | -0.33 | 0.16 |
| 4 | 91.71 | 1.52 | 0.49 | -0.25 | 0.23 | -0.53 | 0.39 | 0.11 | -0.30 | -0.34 |
| 5 | 59.91 | 1.89 | -0.18 | -0.65 | 0.04 | 0.01 | 0.39 | 0.23 | 0.24 | 0.53 |
| 6 | 53.92 | 1.99 | 0.03 | 0.19 | -0.37 | -0.10 | 0.32 | -0.47 | -0.51 | 0.47 |
| 7 | 51.04 | 2.04 | 0.03 | 0.11 | 0.28 | -0.37 | 0.08 | -0.62 | 0.61 | 0.08 |
| 8 | 40.41 | 2.30 | -0.31 | 0.60 | 0.46 | -0.08 | 0.39 | 0.37 | -0.05 | 0.17 |

Table S2. Parameter estimates with 95% confidence limits for size-class interactions (A) and covariate effects (B) for the top two multivariate autoregressive state-space models. Bold values indicate estimates with confidence limits excluding zero (excluding diagonal estimates of density dependence). The effect of size class *i* on itself is *b_i,i_* - 1. Data were standardized with z-scores so that coefficients are comparable across variables.

| 1. Parameter estimates for interactions among variates, **B** | | | | | | | | | | | | |  |
| --- | --- | --- | --- | --- | --- | --- | --- | --- | --- | --- | --- | --- | --- |
|  | Model 1 | | | |  | Model 2 | | | | | | |  |
| Variate | Juvenile | Sub-adult | Adult | |  | Juvenile | | Sub-adult | | Adult | |  |  |
| **Parthenia** |  |  |  | |  |  | |  | |  | |  |  |
| Juvenile | 0.26 [-0.09, 0.51] | -0.09 [-0.47, 0.19] | 0.25 [-0.01 ,0.54] | |  | 0.28 [-0.07, 0.52] | | -0.13 [-0.53, 0.17] | | **0.26 [0.02, 0.58]** | |  |  |
| Sub-adult | **0.44 [0.21, 0.71]** | 0.14 [-0.27, 0.47] | -0.11 [-0.45, 0.18] | |  | **0.45 [0.23, 0.71]** | | 0.12 [-0.32, 0.42] | | -0.11 [-0.41, 0.18] | |  |  |
| Adult |  | 0.03 [-0.36, 0.41] | 0.23 [-0.22, 0.52] | |  |  | | 0.00 [-0.43, 0.36] | | 0.22 [-0.19, 0.53] | |  |  |
| **Eliza** |  |  |  | |  |  | |  | |  | |  |  |
| Juvenile | 0.66 [0.43, 0.77] | -0.08 [-0.25, 0.08] | **0.20 [0.09, 0.35]** | |  | 0.64 [0.43, 0.76] | | -0.07 [-0.23, 0.08] | | **0.18 [0.06, 0.32]** | |  |  |
| Sub-adult | **0.29 [0.19, 0.46]** | 0.67 [0.42, 0.79] | -0.09 [-0.26, 0.04] | |  | **0.27 [0.16, 0.45]** | | 0.68 [0.45, 0.81] | | -0.11 [-0.29, 0.01] | |  |  |
| Adult |  | 0.09 [-0.13, 0.30] | 0.63 [0.38, 0.75] | |  |  | | 0.09 [-0.1, 0.28] | | 0.61 [0.35, 0.73] | |  |  |
| 1. Parameter estimates for the effect of covariates, **C** | | | | | | | | | | | | |  |
|  | Model 1 | |  |  | | | Model 2 | | | | |  |  |
| Variate | Flow Lag | Sediment |  |  | | | Flow Lag | | Sediment | | Algae | | |
| **Parthenia** |  |  |  |  | | |  | |  | |  | | |
| Juveniles | **0.23 [0.11, 0.42]** | **-0.33 [-0.54, -0.17]** |  |  | | | **0.23 [0.11, 0.42]** | | **-0.32 [-0.56, -0.15]** | | 0.06 [-0.11, 0.25] | | |
| Sub-adults/Adults |  | **-0.23 [-0.48, -0.06]** |  |  | | |  | | **-0.24 [-0.46, -0.04]** | | 0.07 [-0.11, 0.27] | | |
| **Eliza** |  |  |  |  | | |  | |  | |  | | |
| Juveniles | **0.16 [0.09, 0.29]** | **-0.18 [-0.30, -0.10]** |  |  | | | **0.13 [0.07, 0.26]]** | | **-0.18 [-0.30, -0.10]** | | **-0.14 [-0.24, -0.04]** | | |
| Sub-adults/Adults |  | -0.01 [-0.13, 0.11] |  |  | | |  | | -0.01 [-0.13, 0.09] | | **-0.12 [-0.24, -0.02]** | | |


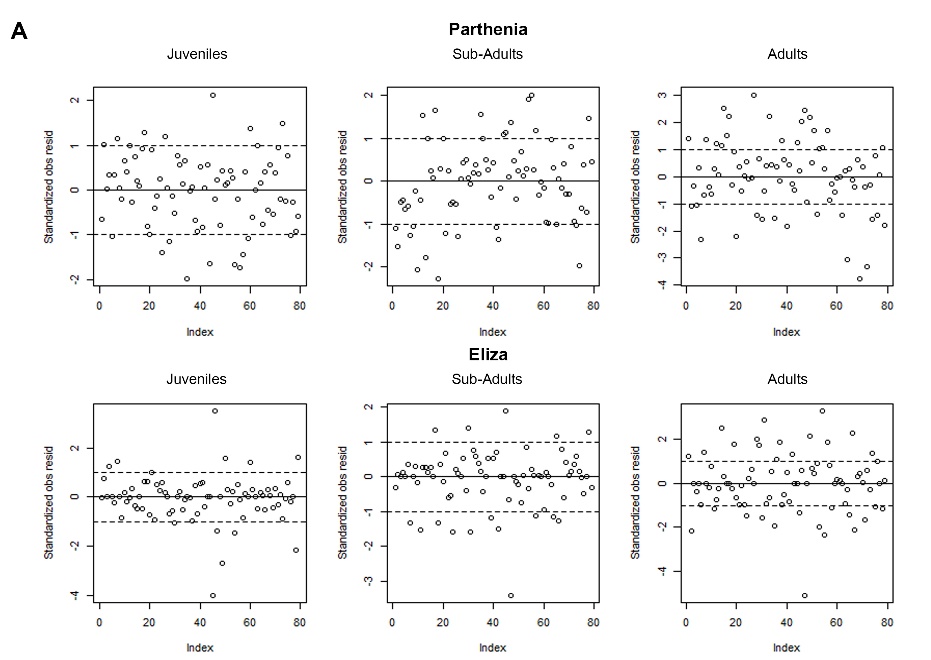

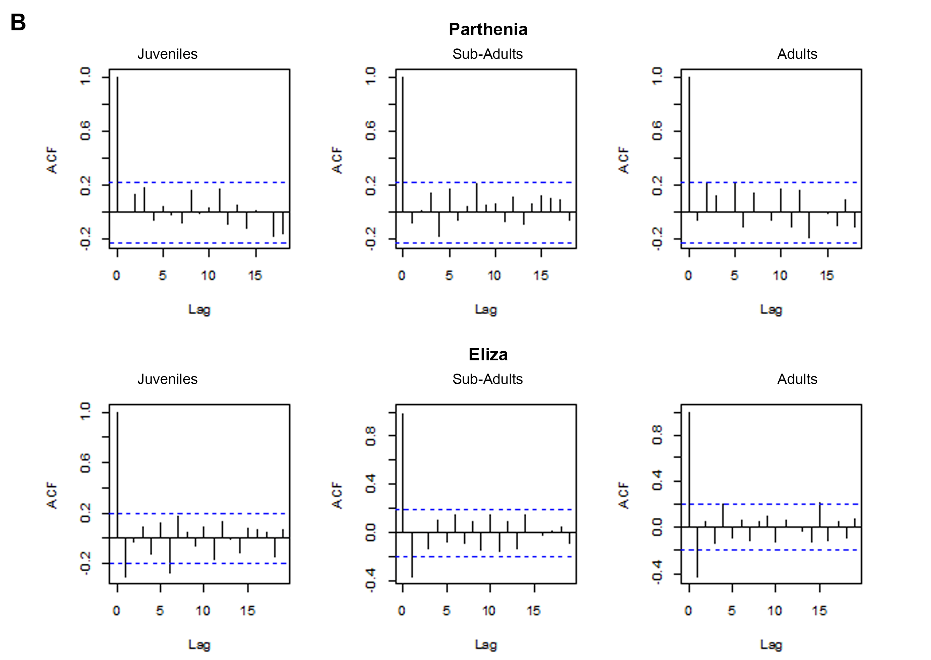


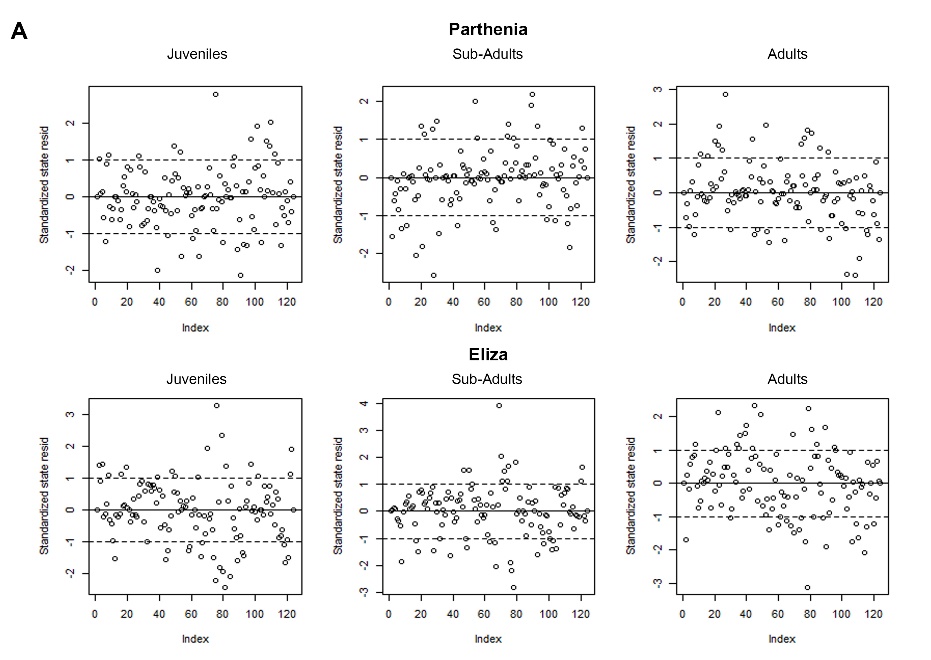

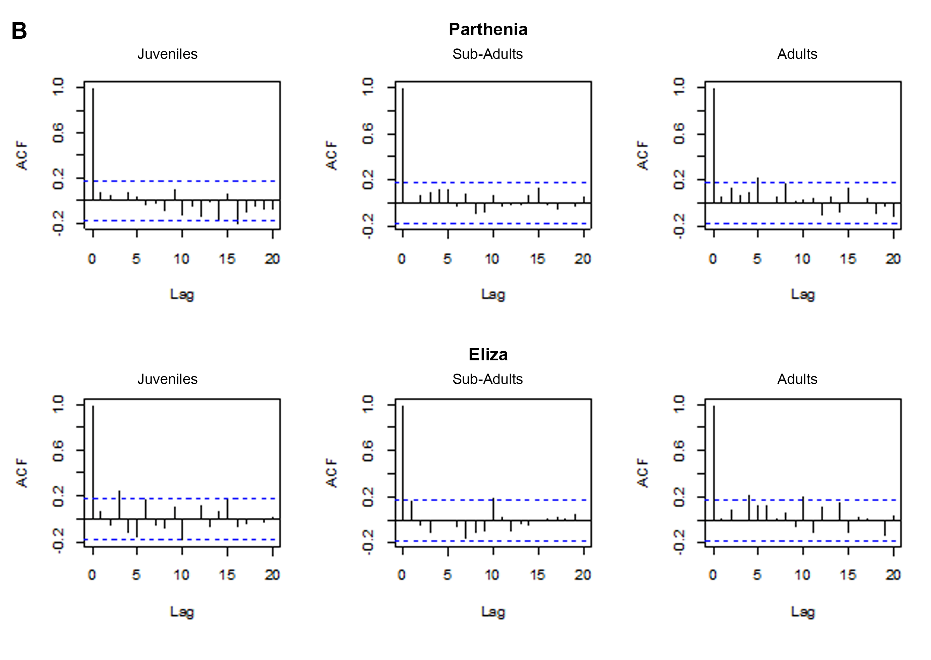
Figure S2. Observation residuals (A) and autocorrelation function (B) plots of observation residuals from the most optimal model.

Figure S3. State residuals (A) and autocorrelation function (B) plots of state residuals from the most optimal model.


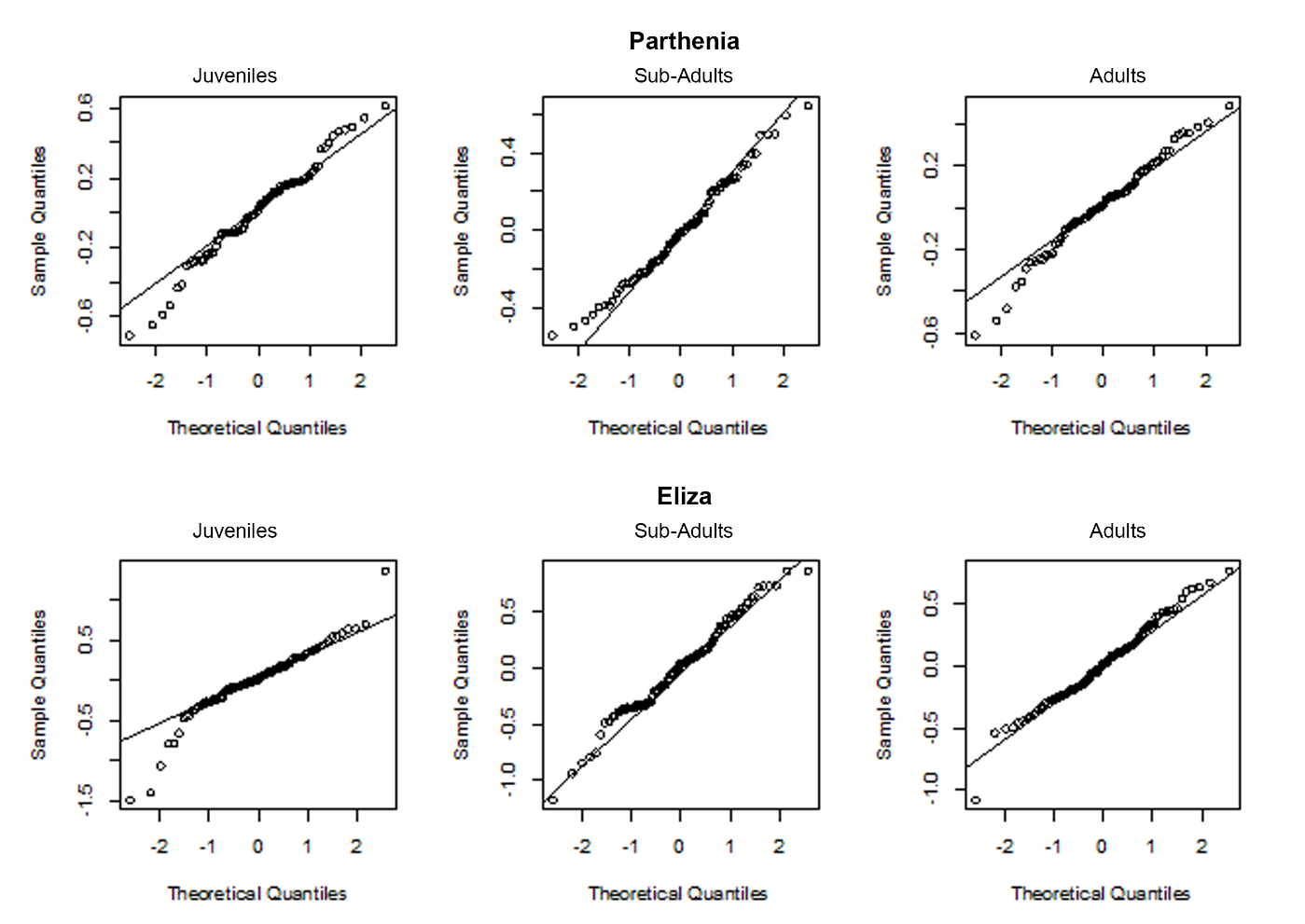


Figure S4. Quantile-quantile plots of the observation residuals.


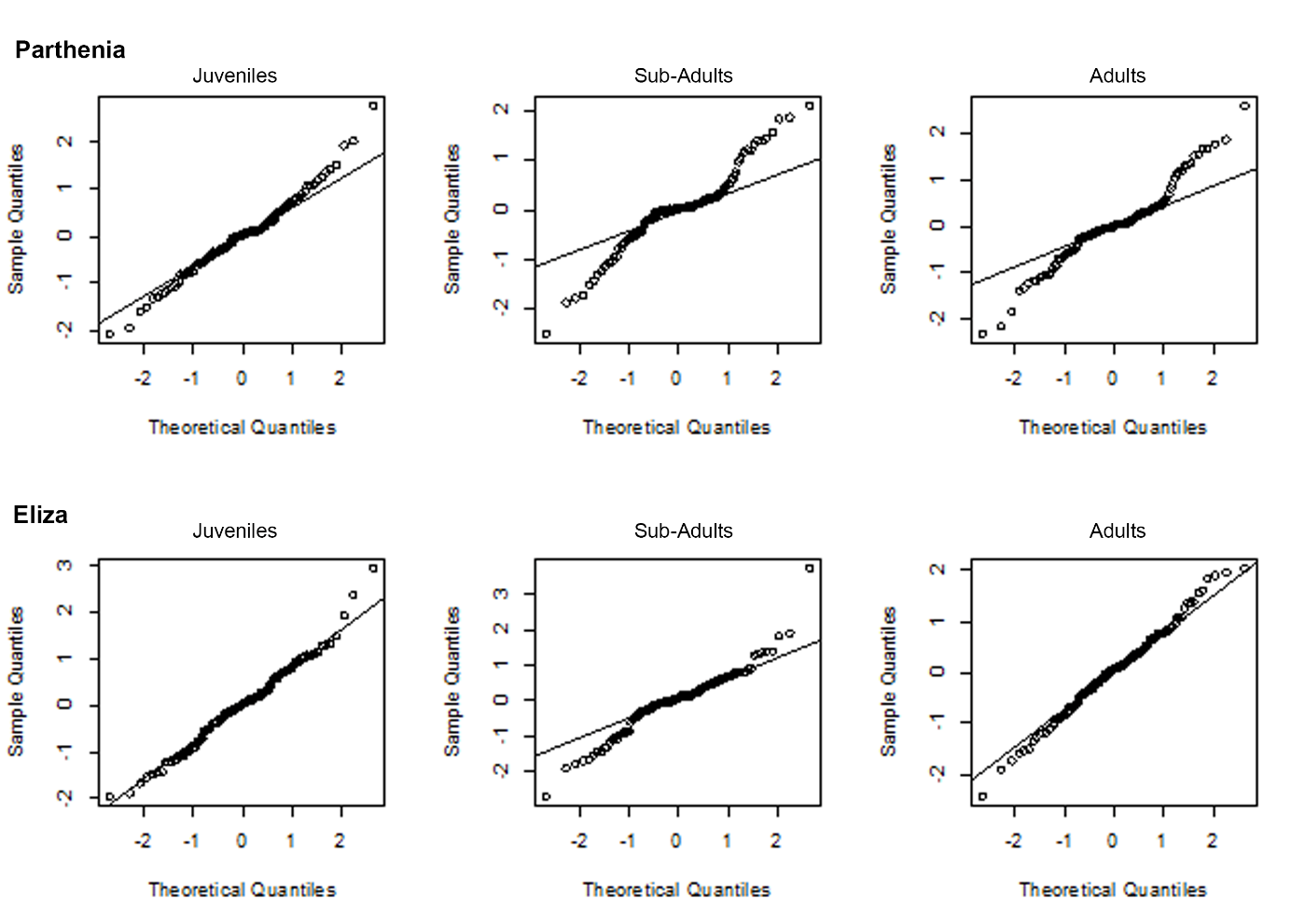


Figure S5. Quantile-quantile plots of the state residuals. Near normality is indicated, with the exception of some points in the upper and lower tails, particularly for the Parthenia sub-adult and adult time series. Ives et al. (2003) point out that normal residuals are not required to have “a well defined MAR(1) process.”


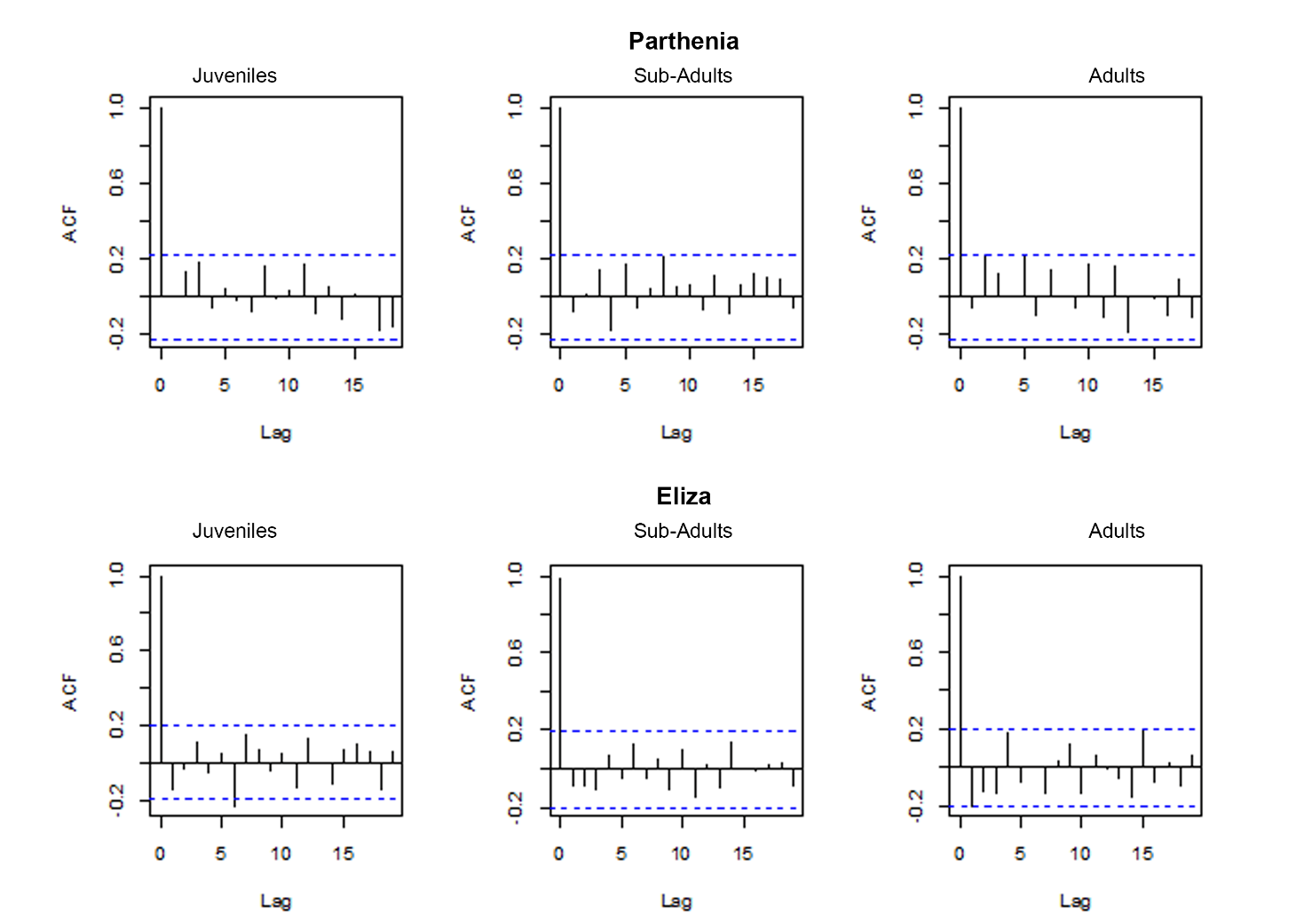


Figure S6. Autocorrelation function (ACF) plots of observation residuals from the most optimal model, with observation error increased. Increasing the observation error eliminates most of the serial autocorrelation in the observation residuals.


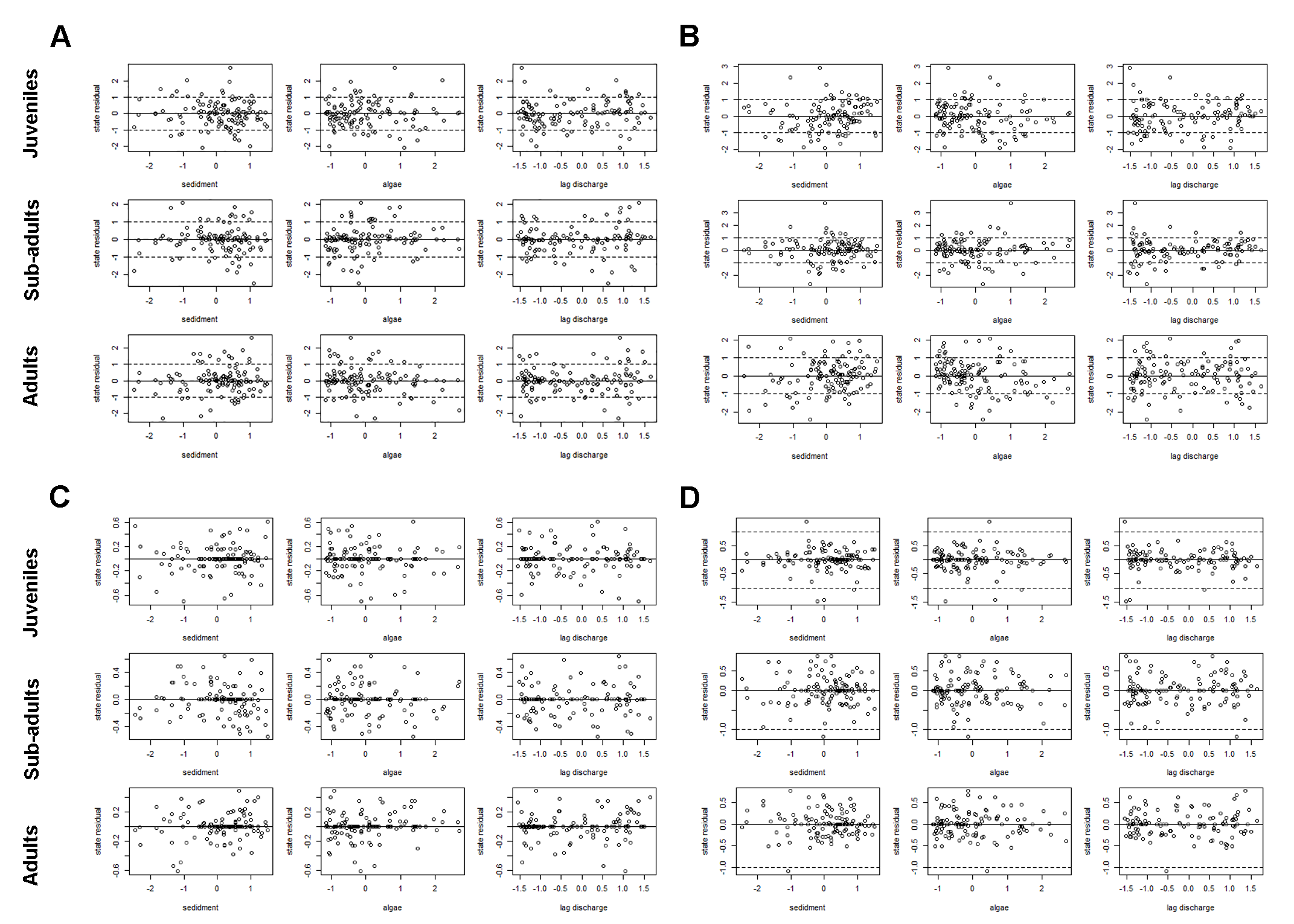


Figure S7. Covariate values (z-scored) vs. state (top row) and observation (bottom row) residuals for Parthenia (A and C) and Eliza (B and D).


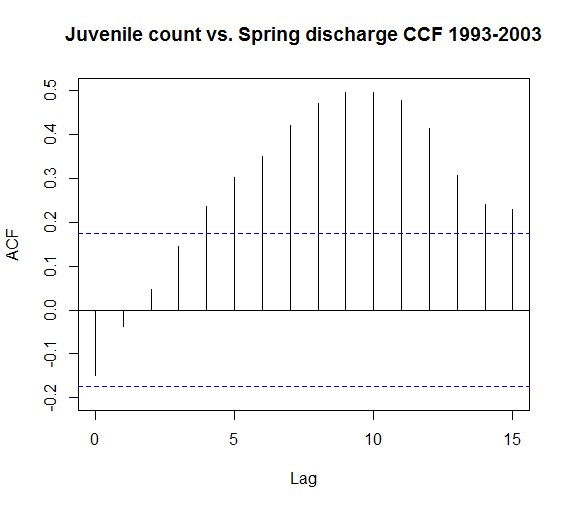


Figure S8. Cross-correlation between counts of juvenile *E. sosorum* collected from 1993–2003 and mean monthly discharge of Barton Springs lagged from 0 to 15 months. Dashed lines represent 95% confidence limits (calculated at lag k = 1).

Table S3. Parameter estimates with 95% confidence limits for size-class interactions (A) and covariate effects (B) for the MARSS model of counts from

1993–2003 at Parthenia.

| **A** | Juvenile | Sub-adult/Adult |
| --- | --- | --- |
| Juvenile | 0.67 [0.31, 0.70] | 0.13 [0, 0.35] |
| Sub-adult/Adult | 0.14 [0.21, 0.71] | 0.76 [0.42, 0.78] |

| **B** | Discharge | Lagged Discharge | Season (cosine) | Season (sine) |
| --- | --- | --- | --- | --- |
| Juvenile | 0.02 [-0.15,0.17] | 0.13 [0.04, 0.35] | 0.09 [-0.08, 0.32] | -0.03 [-0.21, 0.16] |
| Sub-adult/Adult | 0.05 [-0.10,0.22] | 0.00 [-0.14, 0.20] | -0.06 [-0.28, 0.116] | 0.01 [-0.20, 0.20] |
